# S-Layer Glycoproteins and Flagellins: Reporters of Archaeal Posttranslational Modifications

**DOI:** 10.1155/2010/612948

**Published:** 2010-07-20

**Authors:** Ken F. Jarrell, Gareth M. Jones, Lina Kandiba, Divya B. Nair, Jerry Eichler

**Affiliations:** ^1^Department of Microbiology and Immunology, Queen's University, Kingston, ON, Canada K7L 3N6; ^2^Department of Life Sciences, Ben Gurion University, Beersheva 84105, Israel

## Abstract

Many archaeal proteins undergo posttranslational modifications. S-layer proteins and flagellins have been used successfully to study a variety of these modifications, including N-linked glycosylation, signal peptide removal and lipid modification. Use of these well-characterized reporter proteins in the genetically tractable model organisms, *Haloferax volcanii, Methanococcus voltae* and *Methanococcus maripaludis,* has allowed dissection of the pathways and characterization of many of the enzymes responsible for these modifications. Such studies have identified archaeal-specific variations in signal peptidase activity not found in the other domains of life, as well as the enzymes responsible for assembly and biosynthesis of novel N-linked glycans. In vitro assays for some of these enzymes have already been developed. N-linked glycosylation is not essential for either *Hfx. volcanii* or the *Methanococcus* species, an observation that allowed researchers to analyze the role played by glycosylation in the function of both S-layers and flagellins, by generating mutants possessing these reporters with only partial attached glycans or lacking glycan altogether. In future studies, it will be possible to consider questions related to the heterogeneity associated with given modifications, such as differential or modulated glycosylation.

## 1. Introduction

Carl Woese initially defined the third form of life, the Archaea, on the basis of the novel oligonucleotide signatures of their small ribosomal subunit RNA [1–3]. Specifically, by generating phylogenetic trees based on 16S rRNA sequences, Woese clearly showed that Archaea formed a unique group, distinct from Bacteria or Eukarya. However, early analysis also revealed that this unusual group of microbes shared a variety of other characteristics, most notably ether-linked membrane lipids, a variety of unusual cell walls (none of which contained murein), atypical DNA-dependent RNA polymerases and later, their own variation of flagella [4, 5]. Indeed, cell wall composition was one of the very first phenotypical traits of the Archaea considered that allowed for differentiation from Bacteria [6] and was considered in the early days of archaeal research to be “the only useful phylogenetic criterion, other than direct molecular phylogenetic measurement” to distinguish between the two prokaryotic domains [7]. A common feature of many genera of Archaea, found in representatives of all the major lineages, is the presence of an outermost component of the cell envelope termed the surface (S)-layer, comprising protein or often glycoprotein subunits that form a regularly structured array.

In addition to their distinctive cell walls, cell surface structures of Archaea are also unusual [8]. While many, such as cannulae [9] and hami [10] are unique to Archaea, even the more commonly found flagella and pili are unlike their bacterial namesakes [11, 12]. Archaeal flagella are the best-studied of the archaeal appendages and are unusual in many aspects, including the initial biosynthesis of the component flagellins with N-terminal class III signal peptides that are cleaved by a prepilin peptidase-like enzyme. As considered below, these traits are all similar to those found in bacterial type IV pili systems but absent in bacterial flagella systems. 

Both S-layer proteins and flagellins are among the most abundant proteins synthesized by the archaeal cell and both can be isolated with relative ease in substantial amounts for biochemical and structural studies. In Archaea, the majority of these proteins appear to be glycoproteins, mainly containing N-linked glycans, yet sometimes also containing glycans O-linked to threonine residues. In fact, archaeal S-layer proteins, especially those from extreme halophiles, served to identify a novel class of prokaryotic glycoproteins [13–16]. More recently, S-layer proteins and flagellins have been widely used as reporter proteins for the study of a variety of posttranslational modifications in Archaea [17–20], including both class I and class III signal peptide removal, N- and O-glycosylation and lipid modification. Despite these advances, it is only in a very small number of Archaea, including *Haloferax volcanii *and* Methanococcus* species, that genetic studies linking specific genes to a particular posttranslational modification have been performed.

## 2. The S-layer of Haloarchaea andMethanoarchaea

Although found in numerous archaeal species, the S-layers of haloarchaea remain the best studied. Indeed, the first description of a S-layer in Archaea was reported in 1956 when electron microscopic examination of* Halobacterium halobium *(*salinarum*) cells revealed a surface presenting morphological units organized in a hexagonal pattern [21]. Later examination of thin-sectioned haloarchaeal cells revealed the presence of a 17 nm thick cell wall beyond the plasma membrane [22–24]. Blaurock et al. [25] later relied on X-ray diffraction to demonstrate a protein layer laying beyond the haloarchaeal plasma membrane at a distance of 8 nm, with a periplasmic-like space being formed from morphological subunits assuming an “inverted-parabola shape”. Enzymatic iodination of *Hbt. salinarum* surface proteins, together with proteolytic treatment, revealed this surface (S)-layer to contain the S-layer glycoprotein [13]. Kessel et al. [26] next proposed a three-dimensional reconstruction of the *Halobacterium* (later renamed *Haloferax*) *volcanii* S-layer glycoprotein and cell envelope after considering the primary sequence of the *Hbt. salinarum* S-layer glycoprotein [14], as well as the earlier X-ray diffraction data and electron microscopic images of negatively stained cell envelopes. In this model, based on reconstruction to a 2 nm resolution, six S-layer glycoproteins form a 4.5 nm thick dome-shaped pore, with the open center expanding as one approaches the membrane. The deduced C-terminal transmembrane domain of each S-layer glycoprotein is thought to anchor the structure to the membrane, while an O-glycosylated domain of the S-layer glycoprotein lying upstream of the transmembrane domain is proposed to act as a spacer unit, propping up the domed structure. While the forces responsible for maintaining the integrity of such assemblies remain unknown, divalent cations have been shown to be important [13, 26]. Given the use of intact cells maintained in their growth medium and the high degree of sample preservation afforded by the rapid freezing, reconstruction of the *Hbt. salinarum* S-layer through the use of electron tomography offered a more realistic view of this structure [27]. It was thus shown that the *Hbt. salinarum* cell envelope assumes the same basic architecture as does the* Hfx. volcanii * S-layer. Despite similarities in their S-layer architecture, *Hbt. salinarum *and* Hfx. volcanii* cells assume very different shapes, with the former appearing as rods and the latter as indented disks, pointing to factors other than the S-layer as affecting cell shape. 

Like their halophilic counterparts, numerous metha-noarchaeal species, such as members of the genus *Methanococcus*, are also surrounded by a glycoprotein-based S-layer [28]. For many methanogens, study of the S-layer has been relatively limited to identification of the major S-layer component, its response to glycoprotein staining procedures and determination of the lattice symmetry by electron microscopy [29–31]. In the case of *M. voltae*, the S-layer is formed from a 76 kDa protein arranged into an hexagonal lattice with a center to center spacing of 10 nm [32]. While the S-layer protein does not stain positively with the periodic acid Schiff reagent, suggesting the absence of protein glycosylation, subsequent mass spectrometry analysis has shown it to contain a N-linked glycan identical to that found on flagellins in this species [33]. Methodologies were developed to create protoplasts of *M. voltae* by removal and regeneration of the S-layer [34]. The protoplasts could be used for transformation of plasmid DNA either directly or by electroporation [35].

In some instances, such as *Methanothermus fervidus* [36], the S-layer surrounds a sacculus of pseudomurein, a peptidoglycan unique to certain methanoarchaeal species and distinct from bacterial murein. In some *Methanosarcina* species, the cell envelope is thought to consist of a protein-based S-layer surrounded by a rigid cell wall composed of methanochondroitin, a heteropolysaccharide reminiscent of eukaryotic chondroitin [37]. 

A model of the S-layer of *Methanolobus limicola*, formed from glycoprotein subunits, was determined using a variety of microscopy techniques. Using standard electron crystallographic techniques, it was determined that the S-layer had p6 symmetry with a lattice constant of 14.7 nm and a thickness of 4.5 nm [38]. Later examination by scanning tunneling microscopy resulted in a thickness determination of 6.5 nm [39]. As in *Hbt. salinarum*, the subunits are thought to assemble into a dome-shaped structure. Moreover, although no spacer elements have been identified linking the S-layer to the cytoplasmic membrane, negative staining does reveal a narrow space of about 5–10 nm between the two layers [38].

*Methanospirillum hungatei* cells present a very complicated envelope profile. Individual cells are surrounded by a cell wall consisting of an S-layer [40] and then a second unusual outer paracrystalline layer termed the sheath [41], consisting of individual and discrete hoop-like components [42]. The ensheathed cells are then separated from the surrounding environment or from neighboring cells by complicated end or spacer plugs [42]. 

Finally, in *Methanocorpusculum sinense*, examination of the role of the S-layer in cell-shape maintenance and cell division revealed that lattice faults in the normal p6 symmetry of the S-layer appear to be sites of incorporation of new subunits and initiation points for cell division [43].

## 3. Archaeal Flagella Assembly and Composition

Biochemical, genetic, and structural studies performed on flagella from several different archaeal species over the last 2 decades have demonstrated the unique nature of this motility apparatus [5, 44]. Flagella have been reported in all of the major subgroupings of cultivatable archaea, including species of extreme halophiles, haloalkaliphiles, methanogens, hyperthermophiles, and thermoacidophiles [45, 46]. Detailed biochemical, structural and/or genetic studies have been reported in a variety of archaeal genera, including *Methanococcus *[47–49], *Methanospirillum* [50–52], *Halobacterium* [53–56], *Haloarcula* [57], *Haloferax* [58], *Sulfolobus* [59], *Natrialba* [60, 61], *Thermococcus* [62], and *Pyrococcus* [63]. However, the bulk of published work on archaeal flagella is focused on *Halobacterium* and *Methanococcus*; that is, members of the Euryarchaeota, and it is not certain that findings in one organism or even within one of the archaeal domains are applicable to all Archaea or even to members of the other major archaeal domain (i.e., crenarchaeotes). There may be fundamental differences between the two domains; for instance, genes known to be essential for flagellation in *Methanococcus* are not found in crenarchaeotes [46, 64] and even something as fundamental as the presence of the hook may be variable, as hooks have not been observed in the crenarchaeote *Sulfolobus* [65]. 

Archaeal flagella are motility structures involved in swimming and, in the one example where this has been examined in any detail (i.e., *Hbt. salinarum*), the flagella can switch their direction of rotation [66–68]. Other than this superficial commonality, archaeal flagella do not bear other similarities to their bacterial counterparts. For example, there are no homologues of bacterial flagella structural or biosynthetic genes contained in any sequenced archaeal genome [67, 69]. Another fundamental difference between the two prokaryotic flagella organelles may be in the driving force for flagellar rotation. Proton or, more rarely, sodium gradients are used to power bacterial flagellar motion [70], while in the one instance where this has been examined in Archaea, again in *Hbt. salinarum*, flagellar motor rotation depends on ATP [71]. Structurally, archaeal flagella are similar to bacterial type IV pili, surface structures involved in a type of motility across solid surfaces called twitching [72], and, critically, lack a central channel that could allow the passage of subunits through the growing structure for assembly at the distal tip [73–75]. Indeed, the archaeal flagellum has been termed “a bacterial propeller with a pilus-like structure” [75]. Accordingly, archaeal flagella share several commonalities with bacterial type IV pili. Most strikingly, the major subunits of the archaeal flagellum, the flagellins, are made as preproteins with unusual, type IV pilin-like signal peptides (class III signal peptides) that are removed by a specific type IV prepilin signal peptidase homologue (FlaK/PibD; see below) [47, 48, 76]. In addition, both the archaeal flagella system and the type IV pili systems contain a homologous ATPase and a conserved membrane component that may serve as the platform for assembly of the structures [77, 78]. These similarities to type IV pili and the lack of a central channel indicate that assembly of the archaeal flagellum takes place by addition of subunits to the base of the structure, as is also the case in pili growth [45], and fundamentally different from the growth of bacterial flagella, where new subunits are added to the distal end after their passage through the central channel [79]. 

A single *fla *operon encompassing up to thirteen flagella-associated genes has been identified in various flagellated archaea, although the core composition of genes involved in flagellation and their arrangement in the genome can vary in different organisms [44]. Unlike most bacterial species, nearly all the flagellated archaeal species contain multiple (i.e., 2–6) flagellin genes, a rare exception being *Sulfolobus *spp., where only a single flagellin gene is found. Studies show that each flagellin has its own function as deletion of a single flagellin usually results in nonflagellated cells [64, 80, 81]. Interestingly, one of the flagellins forms the hook region in *Methanococcus* species [49, 64, 82] and *Hbt. salinarum* [82]. The *fla* operon typically begins with the multiple flagellin genes, followed by the conserved *fla*-associated genes, *fla*C-*flaJ*, or a subset thereof. The preflagellin peptidase gene is typically located outside this main locus. Deletion analysis has demonstrated that all of the successfully deleted *fla*-associated genes are essential for flagellation, even though some of these genes are not found in all flagellated archaea [64]. Of the *fla*-associated genes, *flaHIJ* are conserved in all flagellated archaea [5]. FlaI and FlaJ are homologous to ATPases (i.e., PilT/PilB) and conserved membrane protein (i.e., PilC/TadB) of type IV pili systems. FlaI has been shown to possess ATPase activity [83]. FlaH may also have ATPase activity, as it contains a conserved Walker box A, although a Walker box B has not been identified [84]. Thus, due to their universal presence in all flagellated archaea and their relationships to type IV pili-related proteins, FlaHIJ are most likely key components in the export and assembly of flagellin subunits. Deletions in any of these genes lead to nonflagellated cells [56, 59, 64, 84, 85]. The roles for the other *fla* associated genes are generally unknown, although recent reports indicate FlaCE and FlaD of *Hbt. salinarum* associate with various Che proteins of the chemotaxis system [86].

Finally, in addition to signal peptide removal, archaeal flagellins also undergo a second posttranslational modification, as most are glycoproteins. While Bacteria sometimes contain glycosylated flagellins, the flagellin glycan is always found in an O-linkage [87]. In Archaea, to date the glycan has always been found to be N-linked to the flagellins [33, 88, 89]. As considered below, the presence and completeness of the glycan has marked effects on the assembly and function of the archaeal flagella. 

In bacteria, flagella do not function only as organelles for swimming but can also be involved in such diverse activities as swarming motility across surfaces, sensing wetness and playing a role in biofilm formation, for example [90–92]. Similarly, the flagella of Archaea have been recently shown to be involved in other important biological functions in addition to their presumably primary role in swimming. In *Pyrococcus furiosus*, flagella can form cable-like connections among cells and in adhesion to *Methanopyrus kandleri* cell surfaces [63]. Interactions of *P. furiosus *with *Methanopyrus *cells can occur through flagella, resulting in a two-component archaeal biofilm [93]. In *Sulfolobus*, in addition to mediating swimming and swarming, flagella, along with pili, have both been shown to be involved in surface adhesion [94]. On the other hand, in *Hfx. volcanii*, flagella were shown not to be involved significantly in surface adhesion [58]. Instead, attachment is mediated by other type IV pilin-like proteins processed by a type IV prepilin peptidase-like enzyme. Swimming without flagella can occur via different mechanisms in bacteria [12] but, to date, no such nonflagellar-driven swimming modes have been reported in Archaea. 

## 4. Posttranslational Modification of S-Layer Glycoproteins and Flagellins

**Figure 1:**
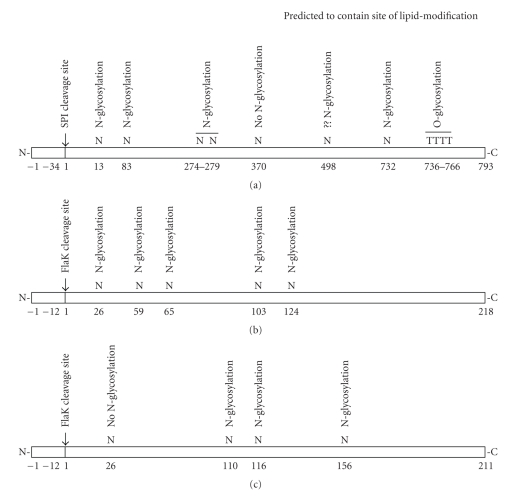
Schematic depiction of the posttranslational modifications experienced by the *Hfx. volcanii* S-layer glycoprotein and *Methanococcus* flagellins. A. *Hfx. volcanii* S-layer glycoprotein; B. *M. voltae *FlaB1; C. *M. maripaludis* FlaB1. Below each sequence, depicted by the elongated rectangle, amino acid residue positions are provided. Above each sequence, the residue at that position is listed, as is the posttranslational modification experienced by that residue or region of the protein. For sequon Asn residues, N-glycosylation, no N-glycosylation or unverified (??) N-glycosylation is marked. Note that the three sequences are not drawn to scale.

In addition to serving important structural and physiological roles, S-layer glycoproteins and flagellins, in particular those from halophilic and methanogen archaea, are important reporters of posttranslational modifications, including signal sequence cleavage, glycosylation and lipid attachment (Figure 1).

### 4.1. Signal Peptide Cleavage

Bacteria can contain as many as three distinct signal peptidases, essential for removing the N-terminal signal peptides that target preproteins for export from the cytoplasm [95]. Signal peptidase I (SPI) is the housekeeping signal peptidase, responsible for cleaving the signal peptides from most preproteins secreted from the cell via either the Sec or TAT pathways. Signal peptidase II (SPII) removes signal peptides specifically from lipoproteins. Finally, type IV prepilin peptidases (TFPP, sometimes termed signal peptidase III, SPIII) are necessary for the cleavage of class III signal peptides from type IV pilins and related molecules. In Archaea, only SPI and TFPP have been identified [18].

**Signal Peptidase I**

Archaeal signal peptidase I was first identified in *Methanococcus voltae*, where the gene was cloned and the protein expressed and studied biochemically *in vitro*, using a heterologously expressed, truncated S-layer protein as substrate [96]. Site-directed mutagenesis studies on the methanogen enzyme and that of *Hfx. volcanii *reached the same conclusion; namely, that the archaeal enzyme relies on a different grouping of essential amino acids than does either the typical prokaryotic (P-type) enzyme found in Bacteria or eukaryotic (ER-type) enzyme [97, 98]. Bacterial P-type SPIs, found as well in mitochondria and chloroplasts, utilize a Ser-Lys dyad for catalysis. Site-directed mutagenesis studies revealed that Ser^90^ and Lys^145^ of *Escherichia coli* SPI are critical for enzymatic activity [99], while subsequent mutagenesis studies, based on crystal structure analysis, lead to the identification of Ser^278^ as being necessary for optimal activity [100]. In the case of ER-type SPIs, no lysine residues are essential for activity, reflecting the use of a different catalytic mechanism. Indeed, the conserved lysine of the P-type SPI is replaced by a conserved histidine in the ER-type SPI [101]. Further mutagenesis studies identified a conserved serine, histidine and two aspartic acid residues as being important for activity of the eukaryal enzyme, suggesting a potential catalytic mechanism relying on a Ser-His-Asp catalytic triad or a Ser-His catalytic dyad [101, 102]. Archaeal SPI maintains the conserved amino acids of the ER-type enzyme, notably the replacement of the conserved lysine by a histidine. However, site-directed mutagenesis again revealed archaeal-specific features. While the essential natures of the conserved serine and histidine residues were demonstrated, only one of the conserved aspartic acid residues was shown to be essential, unlike the yeast SPI, where both are essential [97, 98]. Almost all archaeal SPIs contain these four conserved amino acids [18], such that the mechanism of catalysis likely involves the Ser-His-Asp triad, as in the ER-type enzyme.

In *Hfx. volcanii*, two functional SPIs, that is, Sec11a and Sec11b, were identified, and although both are expressed, only Sec11b was deemed essential [103]. Since the two enzymes cleaved substrates differentially in an *in vitro* assay, they may serve distinct physiological roles.

**FlaK/PibD: Archaeal Type IV Prepilin-Like Peptidases**

The best studied of all archaeal signal peptidases are the TFPPs, represented mainly by FlaK/PibD [48, 76, 104], as well as by EppA [105]. These enzymes were first identified as TFPP homologues, able to cleave class III signal peptides from archaeal flagellins. Unlike bacterial flagellins, archaeal flagellins are synthesized as preproteins containing unusually short type IV pilin-like signal peptides. Such processing is an essential step in the assembly of archaeal flagella, as *flaK* mutants are nonflagellated [76].

Site-directed mutagenesis of both enzymes and substrate has greatly contributed to our knowledge of the mechanism of action and substrate range of archaeal TFPPs. Initial studies involving mutation of conserved amino acids in the signal peptide of model substrates, such as flagellins and sugar-binding proteins, revealed general similarities to the type IV pili system, where glycine at the −1 position of the signal peptide (i.e., the position immediately upstream of the cleavage site) was strongly preferred, with alanine shown to be an acceptable substitute. The −2 and −3 positions of the signal peptide are usually basic amino acids and, in the case of *Methanococcus* FlaK, the presence of lysine at the −2 position of the substrate is critical for cleavage [106]. *Sulfolobus* PibD is much less stringent in this regard, in keeping with the large number of potential substrates processed by this enzyme [104]. As revealed initially by signal peptide analysis of potential substrates [104] and later shown directly by *in vitro* assays [107], PibD is also able to accommodate extremely short signal peptides which are not processed by FlaK. Indeed, it has been suggested that PibD is a rarity among archaeal TFPPs in terms of its range of substrates [46], since, in *Methanococcus* for example, there are no sugar-binding proteins and type IV pilins are processed by a different dedicated TFPP, EppA [105]. Key to the activity of EppA seems to be the glutamine residue at position 1 found in all pilins but not in flagellins, since flagellins modified to include the pilin −2 to +2 amino acid region were cleaved [105]. In keeping with their processing of type IV prepilin-like molecules, site-directed mutagenesis of both FlaK [76] and PibD [108] revealed that the pair of conserved aspartic acid residues that align with two aspartic acid residues shown to be essential for the bacterial type IV prepilin peptidase activity are also essential in the archaeal enzymes. Other conserved aspartic acid residues are not essential. Thus, both the bacterial and archaeal enzymes rely on the same catalytic mechanism and belong to the same family of novel aspartic acid proteases.

In *Methanococcus maripaludis*, both flagella and type IV-like pili are composed of major structural proteins possessing class III signal peptides. Interestingly, this species expresses two TFPPs, with FlaK specifically processing flagellins and EppA specifically cleaving the signal peptides from the prepilins [105]. This is not the case in *Sulfolobus solfataricus*, where PibD has a much broader substrate range, cleaving flagellins and type IV pilins, as well as a number of sugar binding proteins which have been hypothesized to form a pilus-like extension from the cell surface, termed the bindosome [8, 109]. In addition, the Iho670 fibers of *Ignicoccus hospitalis*, representing a novel archaeal surface structure, are also composed of subunits that contain class III signal peptides cleaved by a TFPP [110]. It thus appears that assembly of surface structures throughout the archaeal domain may rely heavily on the type IV pilus-like model.

## 5. N-Glycosylation

While the glycoprotein-based composition of the *Halobacterium* cell envelope had been previously suggested [111, 112], it was Mescher and Strominger [13] who purified and characterized the *Hbt. salinarum* S-layer glycoprotein, at the same time presenting the first example of a noneukaryal N-glycosylated protein. Initial efforts at describing the process of archaeal N-glycosylation revealed similarities to the parallel eukaryal process. In both cases, oligosaccharides are assembled on dolichol lipid carriers and transferred to target proteins following their delivery across a membrane; namely, the ER membrane in Eukarya and the plasma membrane in Archaea (cf. [113]). However, it was only thirty years later, with the availability of complete genome sequences, that delineation of the biochemical pathways of archaeal N-glycosylation began in earnest. Almost simultaneously, *agl* (*a*rchaeal *gl*ycosylation) genes implicated in this posttranslational modification were identified in *Hfx. volcanii* [114] and *M. voltae* [115]. Subsequently, *agl* genes participating in the *Methanococcus maripaludis* N-glycosylation pathway were identified [116, 117].

**Haloferax volcanii**

In *Hfx. volcanii*, *agl* genes implicated in the assembly and attachment of a pentasaccharide to select Asn residues of the S-layer glycoprotein were first identified on the basis of their homologies to known N-glycosylation components in Eukarya or Bacteria (i.e., *Campylobacter jejuni*), the only bacterium for which a complete N-glycosylation pathway has been defined (cf. [118]). Subsequently, additional *agl* genes were identified either based upon their proximity to previously identified *agl* sequences or upon reannotation of that region of the genome where all but one of the previously identified *agl* sequences clustered [119–123]. In this manner, AglJ, AglG, AglI, AglE, and AglD, glycosyltransferases responsible for assembly of the S-layer glycoprotein-modifying pentasaccharide, comprising a hexose, two hexuronic acids, a methylester of hexuronic acid and a final hexose, were identified [123, 124]. AglB was shown to be the oligosaccharyltransferase responsible for delivery of the pentasaccharide, and apparently its precursors, to at least two residues of the S-layer glycoprotein; namely, Asn-13 and Asn-83 [123]. In addition, AglF, AglM, and AglP have also been shown to participate in the assembly of the pentasaccharide [120, 122, 124].

While involvement of each of these gene products in S-layer glycoprotein N-glycosylation has been shown through a combination of gene deletion and mass spectrometry approaches, in several instances, biochemical characterization has been carried out. In the case of AglF and AglM, the proteins have been purified and assays compatible with hypersaline conditions have been developed. Accordingly, AglF was shown to be a glucose-1-phosphate uridyltransferase, able to generate UDP-glucose from glucose-1-phosphate and UTP in a NAD^+^-dependent manner, while AglM was revealed to function as a UDP-glucose dehydrogenase, generating UDP-glucuronic acid from UDP-glucose [122]. Indeed, a coupled reaction containing AglF, AglM, glucose-1-phosphate, UTP and NAD^+^ led to the appearance of UDP-glucuronic acid, thus representing the first step towards in vitro reconstitution of the *Hfx. volcanii* N-glycosylation process. In the case of AglP, purification and subsequent development of an *in vitro* assay to test the function of the protein confirmed AglP to be a S-adenosyl-L-methionine-dependent methyltransferase [124], as predicted by earlier bioinformatics analysis [121]. Specifically, AglP acts on the fourth subunit of the pentasaccharide decorating the *Hfx. volcanii* S-layer glycoprotein, adding a methyl moiety to a hexuronic acid to yield the 190 Da methyl ester of hexuronic acid found at this position [124]. These results are summarized in Table 1.

**Methanococcus voltae**

The N-linked glycan described for *M. voltae* PS is a trisaccharide component with structure *β*-Man*p*NAcA6Thr-(1–4)-*β*-Glc*p*NAc3NAcA-(1–3)-*β*-Glc*p*NAc [33], although a strain harbouring a tetrasaccharide variant of this (the same trisaccharide as above with an extra 220 or 260 Da moiety attached) has been reported [125]. The glycan is linked to select asparagine residues present in flagellins and S-layer components via an N-acetylglucosamine, rather than the hexose observed in *Hfx. volcanii*. A combination of techniques, including insertional inactivation of targeted genes, immunoblot, heterologous expression studies and mass spectrometry analysis of purified flagella have identified the glycosyltransferases and the oligosaccharyltransferase required for the assembly and attachment of the glycan. AglH and AglA are responsible for the addition of the first and last sugar residues to the trisaccharide, respectively, [115, 126]. The role of AglH was elucidated by its ability to successfully complement a conditionally lethal mutation in *alg7* (N-acetylglucosamine-1-phosphate transferase) in yeast. AglC and AglK have both been implicated in the transfer of the second sugar residue [125]. Mutants in the oligosaccharyltransferase (*aglB*) contain S-layer glycoproteins and flagellins presenting molecular masses smaller than observed in any of the other *agl* mutants, consistent with this enzyme being responsible for the transfer of the N-glycan. The viability of strains carrying a disruption of *aglB* indicates that the N-linked glycosylation pathway is not essential in *M. voltae* [115].

**Methanococcus maripaludis**

With the development of advanced genetic tools [127], elucidation of the N-linked glycosylation pathway in methanogenic archaea has continued in *M. maripaludis, *where a tetrasaccharide glycan is N-linked to flagellin subunits [88]. The reported structure of the N-linked glycan was Sug-4-*β*-ManNAc3NAmA6Thr-4-*β*-GlcNAc3NAcA-3-*β*-GalNAc, where Sug was a previously unreported  (5*S*)-2-acetamido-2,4-dideoxy-5-*O*-methyl-*α*-l-*erythro*-hexos-5-ulo-1,5-pyranose, representing the first example of a naturally occurring diglycoside of an aldulose [88]. Although the glycans of the two *Methanococcus* species are related, an obvious difference is that *M. maripaludis* uses N-acetylgalactosamine as the linking sugar, as compared to N-acetyglucosamine in *M. voltae*. In addition, the third sugar in both species is the same, except for a 3-acetamidino group addition in *M. maripaludis* that is carried out by the product of *MMP1081* (K. F. J., unpublished results). Interestingly, a strong homologue of MMP1081 is also found in the sequenced genome of *M. voltae *A3. Should this gene also be present in *M. voltae *PS; namely, that strain used for glycan structural study, it is unclear why an acetamidino group would not also be added here.

The genes *MMP1079*, *MMP1080* and *MMP1088*, designated *aglO*, *aglA* and *aglL*, respectively, have been implicated by deletion/complementation analysis and mass spectrometry as being the glycosyltransferases responsible for transfer of the second, third and fourth sugars to the glycan structure, respectively, [116]. As in *M. voltae,* but unlike the case in *Hfx. volcanii*, where all but one of the *agl* genes are found in one large cluster, a*glB *is located elsewhere on the *M. maripaludis* chromosome. Its deletion leads to the appearance of nonglycosylated flagellins [116]. The glycosyltransferase responsible for the transfer of the first sugar residue has yet to be identified. Interestingly, mutants harboring deletions in genes that lead to a nonflagellated phenotype (i.e., *aglB* and *aglO*) initially synthesize normal levels of the flagellins and other cotranscribed *fla* gene products. However, upon continued laboratory sub-culturing, these strains appear to stop transcription of the entire *fla* operon. Other genes identified as involved in the glycan synthesis include *MMP0350,* the product of which is likely responsible for addition of one of the two acetyl groups found on the second sugar [117] and *MMP1085* which encodes a protein responsible for attachment of the methyl group to the terminal sugar (K. F. J. unpublished results). Available information on N-glycosylation in the two *Methanococcus* species is summarized in Table 2.

In addition to the genetic studies described, heterologous expression and in vitro biochemical and enzymatic studies of proteins predicted to be involved in the glycosylation pathway have helped described the biosynthesis of the acetamido sugar subunit precursors in methanococci [128].

## 6. O-Glycosylation

In addition to N-glycosylation, the *Hbt. salinarum* and *Hfx. volcanii* S-layer glycoproteins also undergo O-glycosylation. In each case, a Thr-rich region upstream of the predicted membrane-spanning domain of the protein is decorated at numerous positions by galactose-glucose disaccharides, linked through the galactose subunit [13, 129]. Essentially nothing is presently known of the archaeal O-glycosylation process.

## 7. Lipid Modification

In addition to signal peptide cleavage and glycosylation, haloarchaeal S-layer glycoproteins also experience covalent posttranslational attachment of lipids. This was first shown when *Hbt. salinarum* cells were incubated with [^3^H]-mevalonate and other tritiated lipid tracers, leading to selective incorporation of the radiolabel into the S-layer glycoprotein [130]. The linked radioactive moiety was subsequently revealed by mass spectrometry to be a novel diphytanylglycerol phosphate. Although the precise location of the attached lipid has yet to be defined, a 28 kDa trypsin-generated fragment derived from the C-terminal region of the protein (residues 731–816) was shown to contain the linked group. In terms of attachment of the lipid, it is thought that phosphodiester-based linkage to either a S-layer glycoprotein Ser or Thr residue is responsible. Hence, it would appear that in addition to the single membrane-spanning domain located close to the C-terminus of the haloarchaeal S-layer glycoprotein, deduced from primary sequence analysis, a lipid moiety also anchors the protein to the membrane. Moreover, given sequence similarities in the same C-terminal region of the *Hbt. salinarum*, *Hfx. volcanii* and *Haloarcula japonica* S-layer glycoproteins [14, 129, 131], it is likely that the latter two similarly experience lipid modification [130]. Indeed, such lipid modification has been demonstrated in the case of the *Hfx. volcanii* S-layer glycoprotein [132, 133].

## 8. Importance of Flagellin and S-Layer Glycoprotein Posttranslational Modifications

The ability to generate deletion mutants of *Hfx. volcanii*, *M. voltae* and *M. maripaludis* as well as the availability of other molecular tools have allowed for the importance of posttranslational modifications of reporter proteins in these species to be addressed.

**Haloferax volcanii**

In *Hfx. volcanii*, the use of deletion strains has provided considerable insight into the importance of N-glycosylation to the cell. Strains lacking the ability to perform N-glycosylation, due to the absence of the oligosaccharyltransferase, AglB, or only able to partially recruit the N-glycosylation pathway, due to an absence of other Agl proteins, present various phenotypes, including an S-layer of modified architecture showing increased susceptibility to proteolytic digestion, enhanced S-layer glycoprotein release into the growth medium, and slower growth in medium of increasing salt [119, 120, 122, 123]. Indeed, differential transcription of the various Agl proteins in response to differing growth conditions, reflected by reverse transcription or real time PCR, points to N-glycosylation as being an adaptive process in *Hfx. volcanii*.

Studies addressing the biogenesis of the *Hfx. volcanii* S-layer glycoprotein have also provided insight into the importance of lipid modification. Metabolic [^35^S] pulse-chase radiolabelling, together with the use of the ribosome-targeted antibiotic, anisomycin, revealed the S-layer glycoprotein to undergo a posttranslational maturation step on the outer surface of the plasma membrane, reflected as an increase in the hydrophobicity and apparent molecular weight of the protein [133]. Support for lipid modification as being responsible for S-layer glycoprotein maturation came from experiments showing that growth in the presence of [^3^H] mevalonic acid led to radiolabel being incorporated into the S-layer glycoprotein and that mevinolin, an inhibitor of 3-HMG-CoA reductase (responsible for converting acetyl-CoA into mevalonic acid), prevented the maturation of the S-layer glycoprotein [132].

Moreover, such lipid modification-based maturation does not occur in the absence of Mg^2+^ [133], required for maintaining haloarchaeal S-layer integrity [13, 26]. As the *Hbt. salinarum* S-layer glycoprotein also undergoes a similar lipid-based maturation step [132], this posttranslational modification may be common to S-layer glycoprotein biogenesis in other haloarchaea.

**Methanococcus voltae and Methanococcus maripaludis**

The flagellins expressed by both *M. voltae *and* M. maripaludis *undergo two major posttranslational modifications necessary for their correct assembly into flagella, namely signal peptide cleavage and N-linked glycosylation. Disruption or deletion of the signal peptidase gene (*flaK)* results in cells that are no longer able to assemble flagella, with the unprocessed flagellins remaining in the cytoplasmic membrane [76]. In the case of *M. maripaludis*, these cells are, however, still piliated, since the type IV pilin-like proteins are processed by a separate prepilin peptidase-like enzyme, EppA [105]. Deletion of *eppA* in a cell that is already deleted for *flaK* results in the appearance of nonflagellated and nonpiliated cells (K. F. J., unpublished results).

Early studies pointed to a critical role for glycosylation in flagella structure in *Methanococcus*. When incubated with bacitracin, a known inhibitor of glycosylation, *Methanococcus deltae *cells became nonflagellated, accompanied by a decrease in apparent molecular weight of the flagellins, suggestive of under-glycosylation [134]. More recently, in both *M. voltae* and *M. maripaludis*, it was shown that at least a two sugar-member glycan must be attached to flagellin subunits for proper assembly of the protein into flagella filaments [115, 116]. Deletion of genes involved in the N-glycosylation pathway of both *M. voltae *and *M. maripaludis *result in flagellin subunits that migrate faster on SDS-PAGE, with the enhanced migration corresponding incrementally to the degree of truncation of the glycan [115, 116]. Motility assays using semisolid agar demonstrated that strains harboring deletions of genes in this pathway that are still able to assemble flagella displayed impaired swimming capabilities, as compared to cells able to produce the native N-linked glycan [116]. Finally, deletion of a single gene, that is, *MMP0350*, assigned as an acetyltransferase necessary for the biosynthesis of the second sugar of the *M. maripaludis* N-linked glycan, resulted in defects in both flagellation and piliation. Since the glycan consisted of only a single sugar in this mutant, the fact that the cells were nonflagellated was not unexpected. However, further examination revealed that while these mutants were generally nonpiliated, apparently intact pili were found in the culture supernatants, indicating that a defect in pili anchoring had occurred [117].

## 9. Conclusions

As the research spotlight begins to shift from the genome to the proteome, it is becoming clear that numerous archaeal proteins experience posttranslational modifications [135–139]. As discussed here, the availability of well-characterized reporters of protein processing events, such as haloarchaeal and methanoarchaeal S-layer glycoproteins and flagellins, offer excellent models in studies attempting to dissect the pathways responsible for such modifications. Along with the identification of additional reporter proteins, it will be possible to consider questions related to the heterogeneity associated with a given modification, such as differential or modulated glycosylation. Moreover, with the development of appropriate *in vitro* assays for these novel reporters, future efforts can address the importance of posttranslational modifications to enzyme function, stability and other traits.

## Figures and Tables

**Table 1:** Effects of *Hfx. volcanii agl* deletions.

Gene	Role	Effect^1^ of deletion on:						
		*Hfx. *volcanii Growth in high salt	S-layer Assembly	Shedding	Susceptibility to protease	S-layer glycoprotein SDS-PAGE migration	N-linked glycan structure	Reference
*aglB*	OTase^2^	Decreased	No effect	Increased	No effect	Increased	No glycan	[114, 123]
*aglD*	GTase^3^ (sugar 5)	Decreased	Perturbed	Decreased	Decreased	Increased	Tetrasaccharide	[114, 123]
*aglE*	GTase (sugar 4)	No effect	n.d.	n.d.	No effect	No effect	Trisaccharide	[119]
*aglF*	Glucose-1-P uridyltransferase (sugar 3)	n.d.^4^	n.d.	n.d.	Increased	Increased	Disaccharide	[120]
*aglG*	GTase (sugar 2)	n.d.	n.d.	n.d.	Increased	Increased	Monosaccharide	[120]
*aglI*	GTase (sugar 3)	n.d.	n.d.	n.d.	Increased	Increased	Disaccharide	[120]
*aglM*	UDP-glucose dehydrogenase (sugars 2, 3 (4?))	n.d.	n.d.	n.d.	Increased	Increased	Monosaccharide	[122]
*aglP*	Methyltransferase (sugar 4)	n.d.	n.d.	n.d.	Increased	n.d.	Modified tetrasaccharide	[124]

^1^Relative to level detected in parent strain; increased, decreased or no effect.

^2^OTase: oligosaccharyltransferase.

^3^GTase: glycosyltransferase.

^4^n.d.: not determined.

**Table 2:** Effects of *M*.* maripaludis *and* M*.* voltae agl* deletions.

Gene	Role	Effect^1^ of deletion on:				
		Cell flagellation	Motility	Flagellin SDS-PAGE migration	N-linked glycan structure	Reference
*M*.* maripaludis *						
*aglA *	GTase^2^ (sugar 3)	Present	Decreased	Increased	Disaccharide	[116]
*aglB *	OTase^3^	Absent	Non-motile	Increased	No glycan	[116]
*aglL *	GTase (sugar 4)	Present	Decreased	Increased	Modified trisaccharide	[116]
*aglO *	GTase (sugar 2)	Absent	Non-motile	Increased	Monosaccharide	[116]
*MMP0350 *	Acetyltransferase (sugar 2)	Absent	Non-motile	Increased	Monosaccharide	[117]
*MMP1081 *	Acetamidino transfer (sugar 3)	Absent	Decreased	Increased	Modified trisaccharide	unp
*MMP1085 *	Methyltransferase (sugar 4)	Present	n.d.	Increased	Modified tetrasaccharide	unp

*M*.* voltae *						
*aglA *	GTase (sugar 3)	Present	n.d.	Increased	Disaccharide	[115]
*aglB *	OTase	Absent	Non-motile	Increased	No glycan	[115]
*aglC *	GTase (sugar 2)	Absent	n.d.	Increased	Monosaccharide	[125]
*aglK *	GTase (sugar 2)	Absent	n.d.	Increased	Monosaccharide	[125]

N. B.: *M. maripaludis* wild type N-linked glycan is a tetrasaccharide, *M. voltae* wild type N-linked glycan is a trisaccharide.

^1^Relative to level detected in parent strain.

^2^GTase: glycosyltransferase.

^3^OTase: oligosaccharyltransferase.

^4^n.d.: not determined.

^5^unp: unpublished data.
